# Test volume data for 51 most commonly ordered laboratory tests in Calgary, Alberta, Canada

**DOI:** 10.1016/j.dib.2019.103748

**Published:** 2019-03-07

**Authors:** Irene Ma, Maggie Guo, Cheryl K. Lau, Zane Ramdas, Rhonda Jackson, Christopher Naugler

**Affiliations:** a Department of Pathology and Laboratory Medicine, University of Calgary, Calgary, Alberta, Canada; b Alberta Public Laboratories, Calgary, Alberta, Canada; c Family Medicine, University of Calgary, Calgary, Alberta, Canada; d Community Health Sciences, Cumming School of Medicine, University of Calgary, Calgary, Alberta, Canada

**Keywords:** Laboratory medicine, Pathology informatics, Laboratory utilization management

## Abstract

Data presented in this article include the top 51 ordered laboratory tests in Calgary and surrounding area, Alberta, from January to December 2017. This data set was collected from Calgary Laboratory Service’s Laboratory Information System, and included top 51 tests ordered from community (n = 11, 224, 330), inpatient (n = 2,340,594) and emergency (n = 1,670,062) settings. Test order mnemonic that were not true laboratory tests (eg: “extra PST tube”, “extra tube”, etc.) were excluded in the analysis. The top test ordered in all 3 test encounters was the complete blood count test (community encounter, n = 921, 873; inpatient setting, n = 357, 375; and emergency setting, n = 276, 954). This data article was submitted as a companion paper to the related research article, “Estimated costs of 51 commonly ordered laboratory tests in Canada” [1].

**Table:** Specifications table

Subject area	*Medicine*
More specific subject area	*Laboratory Medicine*
Type of data	*Table*
How data was acquired	*Calgary Laboratory Services’ Laboratory Information System (LIS)*
Data format	*Raw, analyzed*
Experimental factors	*Calgary Laboratory Services is the sole laboratory services provider for Calgary and surrounding area, with a catchment population of over 1.6 million individuals. All test counts by order mnemonic were collected from the LIS in the 2017 calendar year. The top 100 test counts by order mnemonic and test encounter (community, inpatient, emergency) were collected. The top 100 list was then narrowed down to top 51 tests ordered by testing encounter by authors CN and IM to remove any tests that were not true laboratory tests (see below for details).*
Experimental features	*Test count by order mnemonic and test encounter (community, inpatient, emergency) was collected from LIS and ranked by the top 51 tests ordered in Calgary and surrounding area in the 2017 calendar year. Order mnemonic that are not true tests (eg: “extra PST tube”, “extra tubes”) were excluded in top 51 list.*
Data source location	*Calgary, Alberta, Canada*
Data accessibility	*Data is with this article*
Related research article	Ma, I., Lau, C.K., Ramdas, Z., Jackson, R., and C. Naugler, *Estimated costs of 51 commonly ordered laboratory tests in Canada*. Clin. Biochem, 2019 *“in press”*[1].

**Table:** 

**Value of the data** • This table of top 51 tests commonly ordered in a large Canadian city can serve as a reference for other national and international jurisdictions when performing quality assessments in their clinical laboratories • Data can be used to determine appropriate test ordering practices by physicians within the city of Calgary as well as various jurisdictions in Canada • This table can be used in collaborations between Canadian clinical laboratories to assess and compare appropriate laboratory utilization of commonly ordered tests

## 1 Data

The data presented here includes over 50 commonly ordered laboratory tests by test volume in Calgary and surrounding area, Alberta, from January to December 2017 (Table 1). This data was collected from the Laboratory Information System of Calgary Laboratory Services (CLS) in order to determine the reference median cost (RMC) of most commonly ordered laboratory tests in Canada [1]. In 2017, the top 51 commonly ordered tests resulted in a test volume of 11, 224, 330 tests, 2,340,594 tests, and 1,670,062 tests for the community, inpatient, and emergency setting, respectively. The most commonly ordered test for all 3 test encounters was the complete blood count test, whereas creatinine was the top 3 ordered test in all 3 encounters in 2017. See Table 1 for a complete list of top 51 tests ordered by volume and encounter.

**Table 1:** Top 51 laboratory tests ordered in 2017 in Calgary, Alberta, by community, inpatient, and emergency encounters.

Rank	Community		Inpatient		Emergency	
	Test name	Test volume	Test name	Test volume	Test name	Test volume
1	Complete blood count	921,873	Complete blood count	357,375	Complete blood count	276,954
2	Creatinine	822,960	Electrolyte panel	317,776	Chem panel 7	181,110
3	Thyroid stimulating hormone	704,519	Creatinine	297,676	Creatinine	83,062
4	Electrolyte panel	657,241	Magnesium	118,421	Electrolyte panel	77,347
5	Alanine amino-transferase (ALT)	642,058	Phosphate	88,395	Liver panel (BILT, ALP, ALT, GGT, LD, lipase)	74,287
6	Hemoglobin A1c	577,127	Scan with morphology	88,105	Troponin-T	71,272
7	LDL cholesterol	562,733	Urea	84,260	Prothrombin time (INR)	58,653
8	Ferritin	536,155	Prothrombin time (INR)	83,448	Urinalysis with microscopic if indicated	49,273
9	Urinalysis with microscopic if indicated	487,258	Calcium	59,709	Blood culture	44,039
10	Alkaline phosphatase	305,126	Glucose random	57,442	Urine culture	43,641
11	Gamma glutamyl-transferase (GGT)	276,456	Partial thrombo-plastin Time	55,435	Magnesium	42,081
12	Iron and total iron binding capacity (TIBC)	264,108	Blood Gas Arterial	51,066	Glucose random	40,934
13	Albumin	255,262	Alanine amino-transferase (ALT)	49,597	Scan with morphology	37,517
14	Vitamin B12	244,400	MRSA culture	44,466	Calcium	37,365
15	Glucose random	239,960	Bilirubin total (BILT)	42,972	Urea	33,881
16	Glucose fasting	206,842	Alkaline phosphatase	42,846	Phosphate	32,387
17	Free T4	199,159	Albumin	38,372	Albumin	29,126
18	Calcium	190,420	Gamma glutamyl-transferase (GGT)	34,293	Alanine amino-transferase (ALT)	27,159
19	Urine culture	188,459	Blood culture	32,482	Ethanol	26,925
20	Prothrombin time (INR)	186,613	Lactate dehydrogenase (LD)	28,016	Alkaline phosphatase	23,197
21	Prostate specific antigen	177,252	Urinalysis with microscopic if indicated	24,321	C-reactive protein	23,002
22	C-reactive protein	171,045	Troponin-T	24,307	Bilirubin total (BILT)	22,313
23	Gynecology cytology report	165,135	Surgical pathology report	23,831	Chemistry whole blood panel venous	21,171
24	Chlamydia/gonorrhoeae (GC) test	160,302	Lipase	21,987	Thyroid stimulating hormone	18,097
25	Creatine kinase	129,244	Urine culture	20,609	Gamma glutamyl-transferase (GGT)	17,886
26	Throat beta strep test	129,048	Bilirubin direct	18,011	Lipase	17,833
27	Electro-cardiogram lab	127,545	C-reactive protein	16,921	D-dimer	17,185
28	Urate	125,540	Newborn metabolic screen	16,215	Acetaminophen	16,754
29	Fecal immuno-chemical test	125,372	ABO&Rh instrument	16,084	Blood gas venous	16,150
30	Urea	124,972	Blood gas cord arterial	16,082	ABO&Rh instrument	15,932
31	Albumin random-urine	114,641	Prothrombin time (INR) and partial throm	13,392	Salicylate	15,648
32	Bilirubin total and direct	100,398	Creatine Kinase	12,655	Creatine kinase	14,320
33	Bilirubin total (BILT)	95,035	Protein total	12,560	Osmolality	13,642
34	Microscopy for BV/yeast	82,689	Thyroid stimulating hormone	10,425	Partial thromboplastin time	12,505
35	Magnesium	81,614	Urate	9695	Throat beta strep test	11,871
36	Surgical pathology report	75,266	Electrolyte panel	9576	Carbon dioxide content	11,425
37	T3 free	75,235	Carbon dioxide content	9514	Electrolyte panel	11,405
38	Scan with morphology	69,509	Computer crossmatch	9150	Beta hCG quantitative	9824
39	Phosphate	65,745	Bilirubin neonatal total (<15 days)	8107	Lactate	9427
40	Trichomonas vaginalis test (NAAT)	63,920	Blood Gas Capillary	7851	NT proBNP	8719
41	Lipase	59,948	Aspartate amino-transferase (AST)	7691	Chlamydia/gonorrhoeae (GC) test	8341
42	Aspartate amino-transferase (AST)	57,766	Vancomycin trough	7405	Lactate dehydrogenase (LD)	8064
43	Lactate dehydrogenase (LD)	48,150	VRE/MRSA Culture	6166	Urinalysis with Microscopic	7793
44	Tissue trans-glutaminase antibody IgA	46,363	Hemoglobin A1c	5902	Prothrombin time (INR) and partial throm	7747
45	Follicle stimulating hormone	43,677	Anaerobic Culture	5894	Sodium	6837
46	Beta hCG quantitative	42,749	Blood gas central venous	5852	Troponin I	6427
47	Estradiol	42,019	Ionized calcium PF	5839	Wound culture	6274
48	Testosterone total	41,919	Calcium ionized	5763	Vitamin B12	6230
49	Luteinizing hormone	38,707	Sodium	5694	Sedimentation rate	6155
50	Hepatitis B surface antibody	38,065	Electrolyte panel random-urine	5590	Hemoglobin A1c	5610
51	Anti-nuclear antibody (ANA)	36,731	Ferritin	5353	Electro-cardiogram lab	5265
Total volume	–	11,224,330	–	2,340,594	–	1,670,062

## 2 Experimental design, materials, and methods

Calgary Laboratory Services (CLS) is the sole supplier of laboratory services for Calgary and surrounding area in Alberta, Canada, performing on average 29 million tests per year for a population of approximately 1.6 million individuals. The study consisted of any laboratory test ordered between January 1, 2017 and December 31, 2017 in the Calgary Zone, a health region that consists of the city of Calgary and surrounding area, Alberta. The Laboratory Information System (LIS) of CLS was accessed to collect test order mnemonics, and test encounters (community, inpatient, and emergency settings). The top 51 laboratory tests ordered by test volume was then ranked from most to least ordered by test encounter (Table 1). This table was then used to determine the reference median cost (RMC) of over 50 most commonly ordered tests across Canada [1]. Any order mnemonic that was not a true test (for example, “extra PST tube”, “extra tube”, etc.) were excluded in the top 51 ranking.
